# Three new species and one subspecies of the *Amynthas corticis*-group from Guangxi Zhuang Autonomous Region, China (Oligochaeta, Megascolecidae)

**DOI:** 10.3897/zookeys.884.30988

**Published:** 2019-10-30

**Authors:** Yan Dong, Michelle Man Suet Law, JiBao Jiang, JiangPing Qiu

**Affiliations:** 1 School of Agriculture and Biology, Shanghai Jiao Tong University, Shanghai, China Shanghai Jiao Tong University Shanghai China; 2 Department of Biology, Hong Kong Baptist University, Hong Kong, China Hong Kong Baptist University Hong Kong China

**Keywords:** Earthworm, cytochrome c oxidase subunit I gene, new species

## Abstract

Three new species and one subspecies of the genus *Amynthas* are described from Guangxi Zhuang Autonomous Region, China: The new species are: *Amynthas maximus* Qiu & Dong, **sp. nov**. and *Amynthas tortuosus* Qiu & Dong, **sp. nov.**, and *Amynthas shengtangmontis* Dong & Jiang, **sp. nov.**, the subspecies is *Amynthas shengtangmontis minusculus***subsp. nov.** All have four pairs of spermathecal pores in 5/6–8/9, which indicates that they should belong to the *corticis*-group. Their morphological characteristics are compared to other similar species in the *corticis*-group from China and other Asian countries, such as *Amynthas pulvinus* Sun & Jiang, 2013, *Amynthas homosetus* (Chen, 1938), *Amynthas corticis* (Kinberg, 1867), *Amynthas dorsualis* Sun & Qiu, 2013, and *Amynthas carnosus* (Goto & Hatai, 1899). In addition, the results presented are confirmed by the pairwise comparison of COI barcode sequences. The pairwise distances between each new species and the other eighteen *corticis*-group species are greater than 14.7% on average. Furthermore, the pairwise distance between *A. shengtangmontis shengtangmontis* and *A. shengtangmontis minusculus* is 10.7–11.4%.

## Introduction

The genus *Amynthas* Sims & Easton, 1972 is the dominant genus of Megascolecidae in China (Jiang 2016, Zhao 2015) and the *Amynthas corticis*-group consists of a large number of species. Before 1972, only 99 species names had been recorded in the group (Sims and Easton 1972). *Amynthas diffringens* (Baird, 1869), *Amynthas divergens divergens* (Michaelsen, 1892), *Amynthas yunnanensis* (Stephenson, 1912), and *Amynthas heterochaetus* (Michaelsen, 1891) are synonyms of *Amynthas corticis* (Kinberg, 1867) (Blakemore 2004). Since then, 22 more species were reported: two species were described from mainland China (Chen et al. 1975, Chen and Xu 1977), seven species from Hainan Island in China (Sun et al. 2012, 2013), eight species from Taiwan Island (James et al. 2005, Tsai et al. 2001, 2007, 2010, Wang and Shih 2010), and five species from Korea (Hong and James 2001, Hong and Kim 2002).

**Figure 1.**
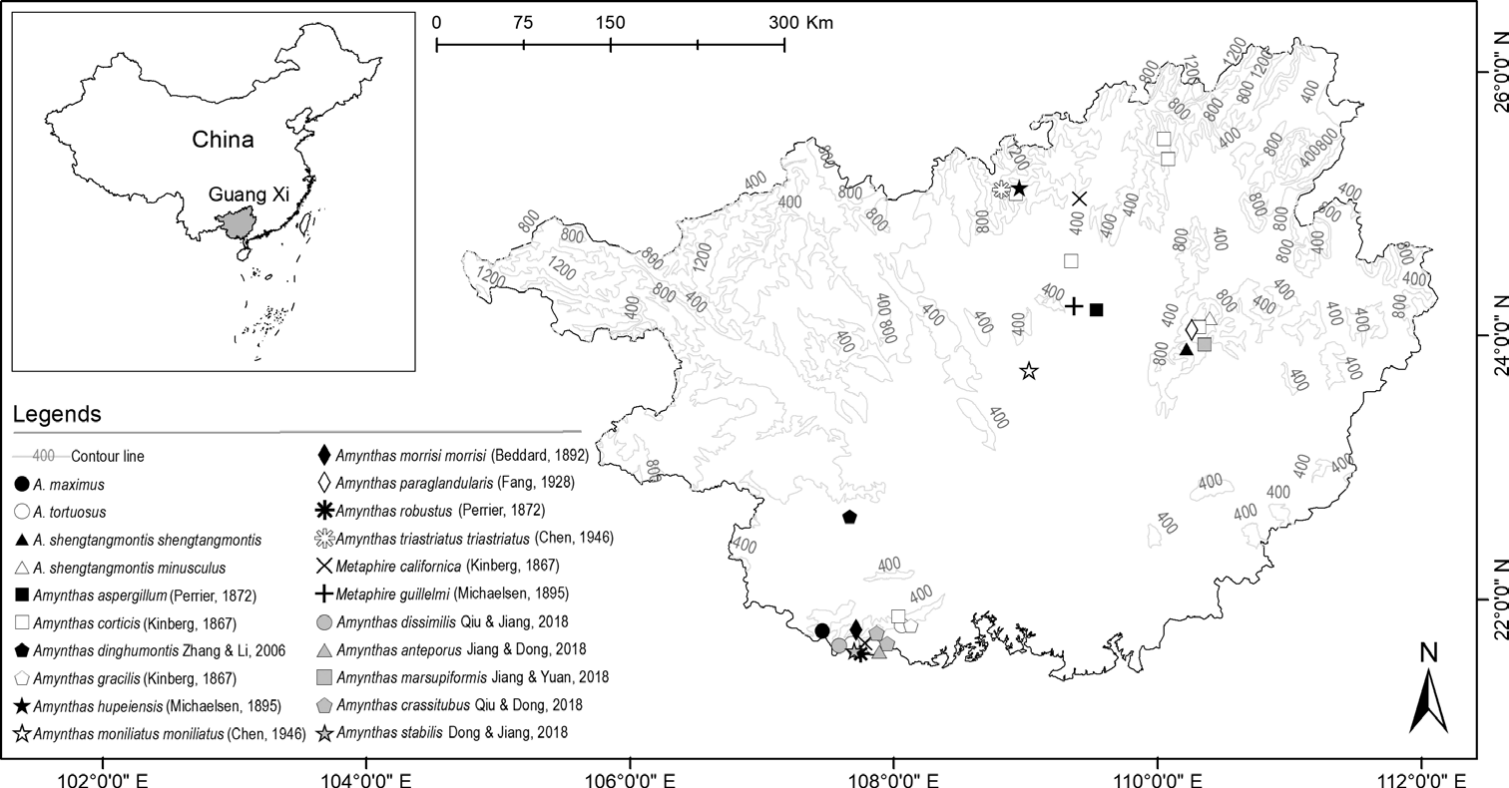
Distribution of known species in Guangxi Zhuang Autonomous Region and sampling points of this paper.

Guangxi Zhuang Autonomous Region is located in the southeast edge of Yunnan-Guizhou plateau, and west of Guangzhou-Guangxi hilly land. The landforms in Guangxi include mountains, hills and plains. Guangxi has a subtropical monsoon climate and the Pearl River, the Yangtze River, the Red River, and the coastal water systems flow through it. Guangxi has an average annual temperature between 16.5–23.1 °C, which is suited to the survival and dispersal of earthworms. In order to investigate the diversity of earthworms in China, we conducted a field survey in Guangxi Zhuang Autonomous Region in 2013 and have found both a number of described species and also species that are new to science. The previously described species are *Amynthas dissimilis* Qiu & Jiang, 2018 (Jiang et al. 2018), *Amynthas anteporus* Jiang & Dong, 2018 (Jiang et al. 2018), *Amynthas marsupiformis* Jiang & Yuan, 2018 (Jiang et al. 2018), *Amynthas crassitubus* Qiu & Dong, 2018 (Dong et al. 2018), and *Amynthas stabilis* Dong & Jiang, 2018 (Dong et al. 2018). In this paper, we describe three new species and a subspecies of *Amynthas* which were collected from the Shiwan Mountain National Nature Reserve (22.6750°~22.07167°N, 107.49972°~108.21972°E) and the Dayao Mountain National Nature Reserve (25.11667°~25.23334°N, 113.18333°~113.26667°E) in Guangxi Zhuang Autonomous Region, China. Distributions of known species in Guangxi Zhuang Autonomous Region and sampling points for this paper are shown in Figure 1.

All of the newly described species and subspecies have four pairs of spermathecal pores in 5/6-8/9; hence, they belong to the *Amynthas corticis*-group.

## Materials and methods

The earthworms were collected in 2013, anaesthetized in 10% ethanol solution, and preserved in 99% ethanol solution. DNA was extracted from several specimens of *A. maximus*, *A. tortuosus*, *A. shengtangmontis shengtangmontis*, and *A. shengtangmontis minusculus* using the EZNA Mollusk DNA Kit (Omega Bio-tek, Norcross, GA, USA). The gene cytochrome oxidase subunit I (COI) was amplified. The PCR was carried out as follows: 5 min at 94 °C followed by 32 cycles 94 °C for 30 s, 50 °C for 30 s and 72 °C for 1 min, with an extension of 10 min at 72 °C. Primers used in the research were: 5'-GGTCAACAAATCATAAAGATATTGG-3'and 5'-TAAACTTCAGGGTGACCAAAAAATCA-3' (Folmer et al. 1994), or 5'-GGTCAACAAATCATAAAGATATTGG-3' and 5'-TATACTTCTGGGTGTCCGAAGAATCA-3' (Bely and Wray 2004). Sequencing was performed in the Beijing Genomics Institute (Shanghai, China). Sequencing was submitted to NCBI GenBank and accession numbers were shown in Table 1. All holotypes and paratypes are deposited in the Shanghai Natural History Museum.

Sequences were aligned with ClustalX (Thompson 1997), and then pairwise distances between these species were calculated using Kimura two-parameter model of DNA evolution with MEGA 5 (Tamura et al. 2011). Images were produced using the Affinity Photo and SketchBook software.

**Table 1.** Species with molecular data used in this study. Abbreviations: HT holotype, PT paratype.

Species	Species No.	Locality	Reference	GenBank Acc. No
*Amynthas maximus* sp. n. (HT)	C-GX201304-01A	China: Guangxi	This paper	MG450707
*Amynthas tortuosus* sp. n. (HT)	C-GX201306-06A	China: Guangxi	This paper	MG450708
*Amynthas tortuosus* sp. n. (PT)	C-GX201301-09	China: Guangxi	This paper	MK606425
*Amynthas tortuosus* sp. n. (PT)	C-GX201305-07	China: Guangxi	This paper	MK606426
*Amynthas shengtangmontis shengtangmontis* sp. n. (HT)	C-GX201312-03A	China: Guangxi	This paper	MG450709
*Amynthas shengtangmontis minusculus* subsp. n. (HT)	C-GX201316-02A	China: Guangxi	This paper	MG450710
*Amynthas shengtangmontis minusculus* subsp. n. (PT)	C-GX201316-02B	China: Guangxi	This paper	MK606427
*Amynthas fuscatus* (Goto & Hatai, 1898)		Japan: Tokyo	Minamiya, submitted to GenBank in 2010	AB542475
*Amynthas pulvinus* Sun & Jiang, 2013	C-HN201115-08	China: Hainan	Sun et al. 2014	JQ905266
*Amynthas robustus* (Chen, 1936)	C-SC201009-01	China: Sichuan	Sun 2013, in Chinese	KF179573
*Amynthas corticis* (Kingberg, 1867)	C-HN201035-02	China: Hainan	Sun 2013, in Chinese	KF205966
*Amynthas carnosus* (Goto & Hatai, 1899)	C-HN201002-01	China: Hainan	Sun 2013, in Chinese	KF205962
*Amynthas mirifius* Sun & Zhao, 2013	C-HN201103-02	China: Hainan	Sun et al. 2013	JQ905265
*Amynthas micronarius* (Goto & Hatai, 1898)		Japan: Tokyo	Minamiya, submitted to GenBank in 2010	AB542498
*Amynthas alexandri* (Beddard, 1900)		Thailand	Jeratthitikul et al. 2017	KU565178
*Amynthas andersoni* (Michaelsen, 1907)		Thailand	Jeratthitikul et al. 2017	KU565179
*Amynthas comptus* (Gates, 1932)		Thailand	Jeratthitikul et al. 2017	KU565187
*Amynthas exiguus* (Gates, 1930)		Thailand	Jeratthitikul et al. 2017	KU565189
*Amynthas formosae* (Michaelsen, 1922)		India	Farooqui, submitted to GenBank in 2019	LC458750
*Amynthas longicauliculatus* (Gates, 1931)		Thailand	Jeratthitikul et al. 2017	KU565195
*Amynthas szechuanensis vallatus* (Chen, 1946)	C-SC201102-05	China: Sichuan	Sun 2013, in Chinese	KF205477
*Amynthas mediocus* (Chen et al., 1975)	C-GD201108-02	China: Guangdong	Sun 2013, in Chinese	KF205405
*Amynthas wulinensis* Tsai et al., 2001		Taiwan island	Chang et al. 2007	DQ224182
*Amynthas yunlongensis* (Chen, 1977)	C-GZ201101-06	China: Guizhou	Sun 2013, in Chinese	KF179581
*Amynthas stricosus* Qiu & Sun, 2012	C-HN201104-04	China: Sichuan	Sun 2013, in Chinese	JX315345

## Taxonomy

### Amynthas maximus

Taxon classificationAnimaliaOpisthoporaMegascolecidae

Qiu & Dong sp. nov.

6A0577B9-A1BE-5C3E-ADE9-2FB3D3446525

http://zoobank.org/E84CFBE5-4FF8-4F53-B49A-233EC5D04298

Figure 2 , Table 2

#### Material.

***Holotype***:1 clitellate (C-GX201304-01A): China, Guangxi Zhuang Autonomous Region, Shiwan Mountain Nature Reserve (21.50299°N, 107.3035°E), 449 m asl, black sandy soil under bryophytes in a subtropical evergreen forest, 12 May 2013, JP Qiu, Y Hong, JB Jiang, LL Zhang, Y Dong legit. ***Paratypes***: 8 clitellates (C-GX201304-01B): same date as for holotype.

#### Diagnosis.

Dimensions 145–170 mm by 5.8–6.2 mm at clitellum, clitellum taupe in 2/5 XIV–XVI, 78–101 segments. First dorsal pore in 13/14. Setae numbering 33–38 at III, 32–36 at V, 29–33 at VIII, 18–22 at XX, 50–65 at XXV; 9–13 between male pores; setae between spermathecal pores numbering 14–16 at VI, 10–14 at VII, and 18–22 at VIII. Four pairs of spermathecal pores ventrally in 5/6–8/9. Seven or eight (three specimens have seven papillae, and four specimens have eight papillae) postsetal genital papillae arranged in two rows in VI–IX, 0.33 circumference ventrally apart from each other. One pair of male pores in XVIII, each on the top of a central, round porophore surrounded by three or four circular ridges, with one presetal indented-topped genital papilla medial of each porophore. Ampulla elongate oval, stout duct as long as 3/5 ampulla. Diverticulum slightly shorter than main pouch, a little twist in the middle, terminal 2/5 dilated into a swollen, club-shaped seminal chamber. Prostate glands well developed.

**Figure 2.**
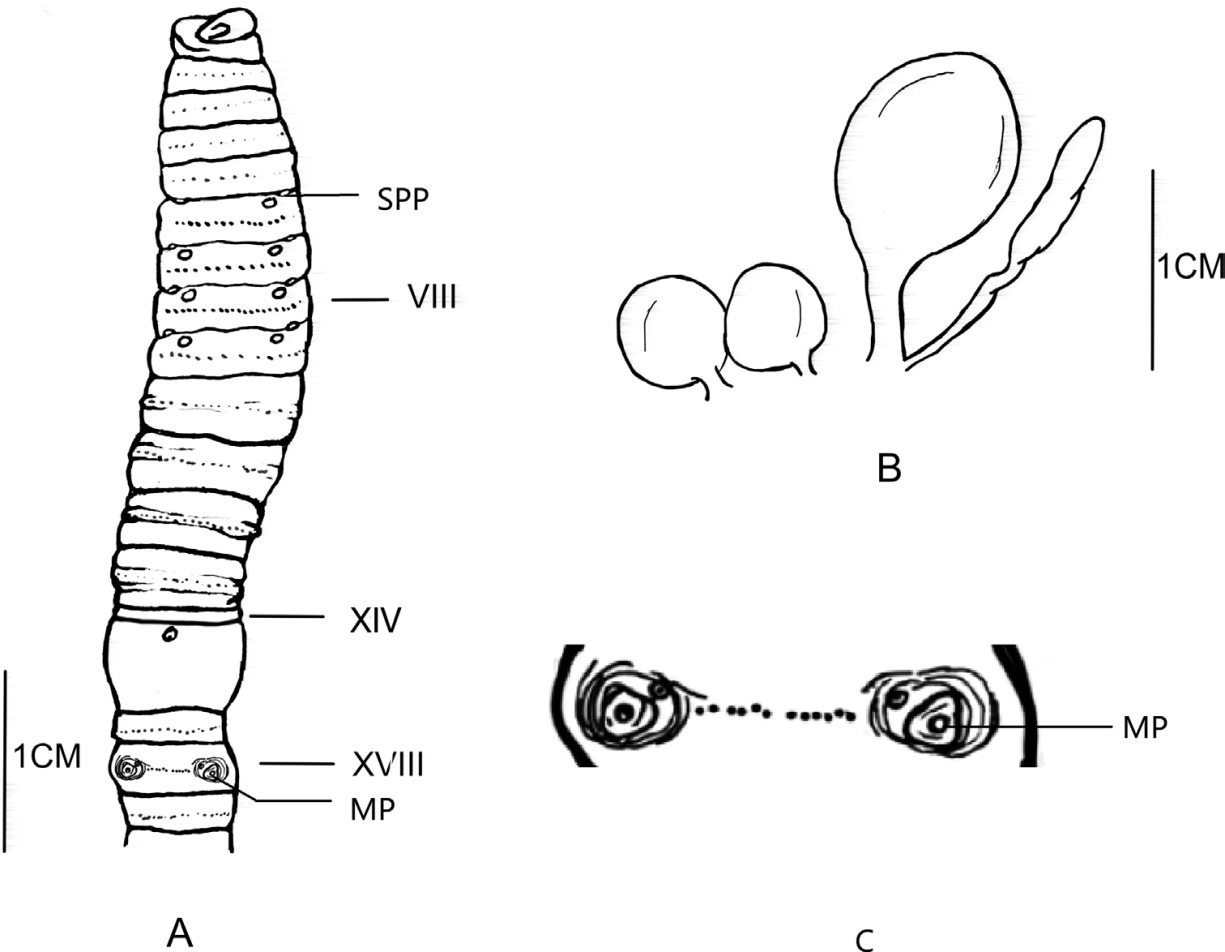
**A** Ventral view showing spermathecal pores, female pores and male pores of *Amynthas maximus* sp. nov. **B** spermathecae of *Amynthas maximus* sp. nov. **C** illustration of the details of the male pore region.

**Table 2.** A comparison of characters of *A. maximus* sp. nov., *A. dorsualis*, 2013, *A. carnosus*, *A. corticis* and *A. wulinensis*.

Characteristics	*A. maximus* sp. nov.	*A. dorsualis*	*A. carnosus*	*A. corticis*	*A. wulinensis*
Body length (mm)	145–170	121–?	110–340	45–170	128–174
Body width (mm)	5.8–6.2	2.7–?	4.0–9.0	3.0–6.0	5.6–6.1
Pigment					
dorsum	Light purple brown before clitellum, from light purple brown to brown after	Dark grey before clitellum, dark brown after	Dark brown or purple	Greenish brown	Whitish purple
ventrum	No pigment before clitellum, yellowish after	Light grey before VII, no pigment after	Dark brown or purple	No	Whitish gray
First dorsal pore	13/14	13/14	12/13	10/11 or 12/13, usually at 11/12	11/12
Clitellum locality	2/5XIV–XVI	1/10XIV-7/10XVI	XIV–XVI	XIV–XVI, occasionally shorter	XIV–XVI
Spermathecal pores	4 pairs, in 5/6–8/9, 0.33C	4 pairs, in 5/6–8/9, dorsally, 0.6C	4 pairs in 5/6–8/9 or 3 pairs in 6/7–8/9, 0.33C	4 pairs, 5/6–8/9, 0.33C	4 pairs, 5/6–8/9, ventral, 0.29C
Male pores	Middle, round, surrounded by 3–4 circular ridges, 0.4C	Slightly raised, glandular, surrounded by 5–6 elliptic circular folds, 0.33C	Round or elliptic	Small, circular to transverse elliptical disc, 0.24–0.30C	Round or oval-shaped on setal line with depressed center, 2–3 circular folds, 0.24–0.28C
Papillae preclitellum	7–8 post-setal indented-topped genital papillae arranged in two rows in VI–IX	Invisible	8 papillae just overhead 8 spermathecal pores, 2 pairs preclitellar arranged on VIII and IX	Paired presetal and/or postsetal in some or all, near spermathecal pores	Absent
Papillae postclitellum	2 presetal indented-topped genital papillae medial of porophore	Invisible	2 paired presetal genital on XVIII and IX, 1 pair postsetal genital on XVIII	Present or absent, occasionally one or more near male pore	Oval-shaped, medial to male pore in each of XVII and XIX, occasionally XX
Prostate glands	XVII–XIX	XVI–XX	Well developed	XVII–XX, rudimentary or absent	XV–XX, racemose, follicular
Spermathecae	About 1.6 mm long, ampulla long-oval; duct as long as 3/5 ampulla	About 2.2 mm long, ampulla heart-shaped; duct as long as 2/5 ampulla	Ampulla oval or pear-shaped, duct equal to or slightly shorter than ampulla	Ampulla ovoid	Very short and stout stalk
Diverticulum	Shorter, lightly twist in middle, terminal 2/5, swollen, club-shaped seminal chamber	Shorter, terminal 1/5, ovoid plump seminal chamber	One-third to half of ampulla, slender stalk and a wider seminal chamber	Blunt ovioid, straight stalk	Oval, shining white seminal chamber, a slender and straight stalk
Accessory glands	1 or 2 stalked accessory glands observed near ventral median line in VI, VII, VIII, IX	Invisible	–	Stalked, coelomic, bound down to parietes or retained within body wall	Paired in XVII and XIX, sessile, flowery

#### Description.

***External characters***: Light purple-brown pigment on pre-clitellum dorsum, no pigment on ventrum. Pigment from light purple-brown to brown on post-clitellum dorsum, light yellowish on ventrum. Clitellum taupe in 2/5 XIV–XVI. Dimensions 160 mm by 6.0 mm at clitellum, 92 segments. Prostomium ½ epilobous. First dorsal pore in 13/14. Setae numbering 36 at III, 34 at V, 31 at VIII, 20 at XX, 60 at XXV; 12 between male pores; Setae numbering 15 at VI, 12 at VII, 20 at VIII between spermathecal pores. Setae formula: AA = 1.1-1.4AB, ZZ = 1.2-2.0ZY. Clitellum annular, yellowish, in 2/5 XIV–XVI, setae not visible externally. Four pairs of spermathecal pores in 5/6–8/9, ventral, eye-like, 0.4 circumference ventrally apart from each other. Seven or eight (three specimens have seven papillae, and four specimens have eight papillae) postsetal genital papillae arranged in two rows in VI–X, 0.33 circumference ventrally apart from each other. One pair of male pores in XVIII, 0.4 circumference apart ventrally, each on the top of a central, round porophore surrounded by three or four circular ridges, with one presetal indented-topped genital papilla medial of each porophore (Figure 1A). Single female pore in XIV, ovoid.

***Internal characters*.** Septa 5/6–7/8, 10/11–13/14 thick and muscular, 8/9–9/10 absent. Gizzard bucket-shaped, in VIII–X. Intestine enlarged distinctly from XV onwards. Intestinal caeca paired in XXVII, extending anteriorly to XXII, transition state, ventral margin smooth, four pointed saccules in dorsal margin. Four pairs of esophageal hearts in X–XIII, developed. Ovaries in XIII. Four pairs of spermathecae in VI–IX, short, approx. 1.6 mm long, ampulla elongate-oval; duct as long as 3/5 ampulla. Diverticulum slightly shorter than main pouch (ampulla and duct), a little twist in middle, terminal 2/5 dilated into a swollen, club-shaped seminal chamber (Figure 1B). One or two stalked accessory glands observed near ventral median line in VI–IX. Holandric: two pairs of testis sacs in X and XI, separated from each other, well developed. Two pairs of seminal vesicles in XI and XII, developed. Prostate glands undeveloped, inserting in XVIII and extending from XVII–XIX, coarsely lobate, prostatic duct I-shaped, of uniform thickness. No accessory glands observed in male pore region.

#### Etymology.

The species is named after its large accessory glands observed in the spermathecal area.

#### Remarks.

*Amynthas maximus* sp. nov. keys to the *corticis*-group in Sims and Easton (1972) with four pairs of spermathecal pores intersegmentally in 5/6–8/9. *Amynthas maximus* sp. nov. is similar to *Amynthas carnosus* (Goto & Hatai, 1899) as re-described by Chang et al. (2016) with respect to body size, the distance between spermathecal pores and male pores, shorter diverticulum than main spermathecal axis, and no accessary glands near prostates. In contrast, the pigment on its ventrum is lighter than *A. carnosus* and other differences include the first dorsal pore, clitellum location, spermathecal pores, and male pores characters. The first dorsal pore in *A. maximus* sp. nov. is located in 13/14, versus 12/13 in *A. carnosus*; the clitellum occupies less than three segments; four pairs of spermathecal pores while sometimes three pairs in *A. carnosus*; the porophore is surrounded by three or four circular ridges, but no ridges are present in *A. carnosus*; several accessory glands observed in the spermathecal region in the new species but none in *A. carnosus*.

We also compare the new species with *Amynthas corticis* (Kinberg, 1867) which has been recognized as the typical species in the *corticis*-group. They share several common characters such as body size, pigment, clitellum extent, setal number, and both have stalked accessory glands. Other than that, the first dorsal pore in the new species is in 13/14, but in 10/11 or 12/13, usually in 11/12 in *A. corticis*. The diverticulum of *A. maximus* sp. nov. has a small twist in the middle compared with *A. corticis*, which has a long stalk. Moreover, *A. maximus* sp. nov. always exhibits genital markings in the male pore region, whereas in *A. corticis*, these markings are occasionally absent.

We further compare the new species with another species *Amynthas dorsualis* Sun & Qiu, 2013 described from Hainan, China, and its clitellum also occupies fewer than three segments. In our results, we find the two species share several common characters, including the first dorsal pore location, setal formula, male pore characters, and in the diverticulum being shorter than the main pouch. However, the morphological dissimilarity of the two species is substantial. For instance, the locations of spermathecal pores are different between *A. dorsualis* and *A. maximus* sp. nov. In *A. dorsualis*, the spermathecal pores are located on the dorsum, while the pores are located on the ventrum in *A. maximus* sp. nov. The distance between male pore is shorter on the ventral side in *A. dorsualis* than *Amynthas maximus* sp. nov. In addition, *A. dorsualis* has no genital markings and no accessory glands, whereas *A. maximus* sp. nov. exhibits genital markings near the spermathecal pores and the male pores region, and stalked accessory glands are present in spermathecal pores region.

The body size of *A. maximus* sp. nov. is similar to *A. wulinensis* described from Taiwan Island. But the other characters of *A. maximus* sp. nov. differ from *A. wulinensis*. *Amynthas maximus* sp. nov. has no pigment before clitellum, yellowish after clitellum on dorsum, first dorsal pore in 13/14, 0.33C between spermathecal pores, seven or eight indented-topped genital papillae in VI–IX and accessory glands in spermathecal pores region. In contrast, *A. wulinensis* has whitish gray on dorsum, first dorsal pore in 11/12, 0.29C between spermathecal pores, no genital papillae observed in spermathecal pores region, and accessory glands observed in male pores region. Table 2 shows the comparison of characters of *A. maximus* sp. nov. with *A. dorsualis*, *A. carnosus*, *A. corticis* and *A. wulinensis*.

### Amynthas tortuosus

Taxon classificationAnimaliaOpisthoporaMegascolecidae

Qiu & Dong sp. nov.

16CA9CE1-D745-575C-8591-9DFB86C165F8

http://zoobank.org/898F2A2C-68E7-415D-B5BE-95876D8C672E

Figure 3 , Table 3

#### Material.

**Holotype**: 1 clitellate (C-GX201306-06A): China, Guangxi Zhuang Autonomous Region, Shiwan Mountain Nature Reserve (21.84739°N, 107.88989°E), 553 m asl, black soil besides road, 13 May 2013, JP Qiu, Y Hong, JB Jiang, LL Zhang, Y Dong legit. **Paratypes**: 7 clitellates: 2 clitellates (C-GX201306-06B): same data as for holotype. 1 clitellate (C-GX201301-09): China, Guangxi Zhuang Autonomous Region, Shiwan Mountain Nature Reserve (21.48588°N, 107.57018°E), 130 m asl, black sandy soil at riverside, 11 May 2013, JP Qiu, Y Hong, JB Jiang, LL Zhang, Y Dong legit. 4 clitellates (C-GX201305-07): China, Guangxi Zhuang Autonomous Region, Shiwan Mountain Nature Reserve (21.50396°N, 107.53350°E), 494 m asl, black sandy soil besides road, 13 May 2013, JP Qiu, Y Hong, JB Jiang, LL Zhang, Y Dong legit.

**Figure 3.**
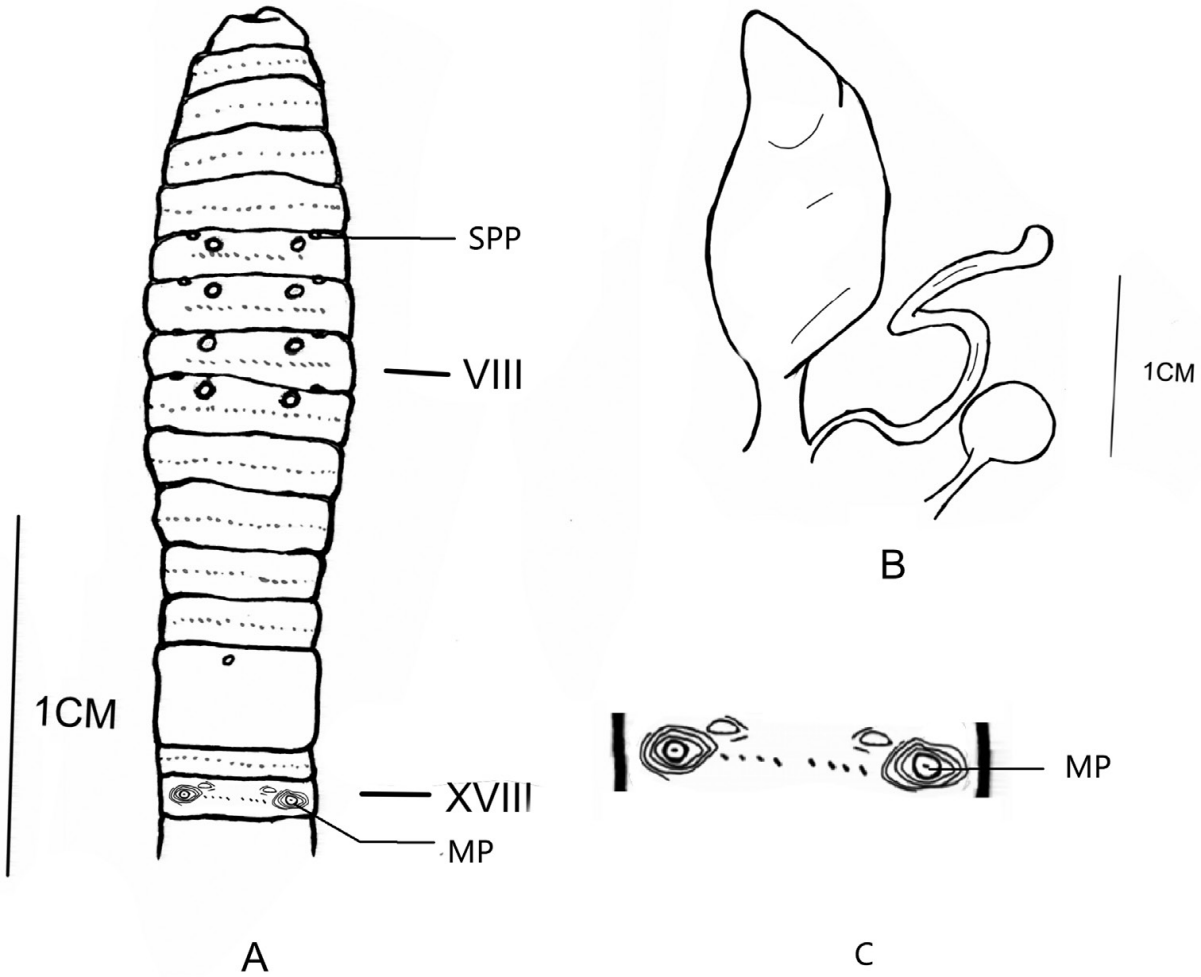
**A** Ventral view showing spermathecal pores, female pores and male pores of *Amynthas tortuosus* sp. nov. **B** spermathecae of *Amynthas tortuosus* sp. nov. **C** illustration of the details of the male pore region.

**Table 3.** A comparison of characters of *A. tortuosus* sp. nov., *A. carnosus*, *A. corticis*, *A. homosetus*, *A. exiguus aquilonius*, and *A. stricosus*.

Characteristics	*A. tortuosus* sp. nov.	*A. carnosus*	*A. corticis*	*A. homosetus*	*A. exiguus aquilonius*	*A. stricosus*
Body length (mm)	55–86	110–340	45–170	116	39–63	72–97
Body width (mm)	2.5–2.8	4.0–9.0	3.0–6.0	5.2	1.9–2.6	2–2.8
Pigment						
dorsum	Purple brown before clitellum, light purple brown after	Dark brown or purple	Greenish brown	Dark chocolate on anterior, grey on other parts	Dark reddish brown	No pigment
ventrum	Light purple brown before clitellum, no after	Dark brown or purple	No	Grey	Light gray on ventrum	No pogment
First dorsal pore	13/14	12/13	10/11 or 12/13, usually at 11/12	12/13	6/7	11/12 or 12/13
Clitellum locality	XIV–XVI	XIV–XVI	XIV–XVI, occasionally shorter	XIV–XVI	XIV–XVI	XIV–XVI
Setal formula	AA=1.2-2.0AB, ZZ=1.4-2.0ZY		–			
Spermathecal pores	4 pairs, in 5/6–8/9, 0.25C	4 pairs in 5/6–8/9 or 3 pairs in 6/7–8/9, 0.33C	4 pairs, 5/6–8/9, 0.33C	4 pairs, in 5/6–8/9, 0.25C	4 pairs, 5/6–8/9, ventral, 0.45C	4 pairs, 5/6–8/9, 0.40C
Male pores	Middle, round, surrounded by 3–4 rhombic ridges, 0.25C	Round or elliptic	Small, circular to transverse elliptical di sc,0.24–0.30C	Roundish glandular area, about 1.5mm in diameter, 0.25C	Round, smooth, slightly elevated with a male aperture inconspicuous on lateral concave area, 0.23–0.30C	on a coniform glandular disc surrounded by a round pad, 0.33C
Papillae preclitellum	Four pairs of postsetal genital papillae in VI–IX	8 papillae just overhead 8 spermathecal pores, 2 pairs preclitellar arranged on VIII and IX	Paired presetal and/or postsetal in some or all, near spermathecal pores	Invisible	presetal and postsetal, widely paired in 7, 8 and 9, number highly variable	Invisible
Papillae postclitellum	2 presetal crescent indented-topped genital papilla medial of male pores	2 paired presetal genital on XVIII and IX, 1 pair postsetal genital on XVIII	Present or absent, occasionally one or more near male pore	Invisible	presetal and postsetal, widely paired in XVII, XVIII and XIX, number highly variable	postsetal, single or paired in XVII, XIX and XX
Prostate glands	XVII–XXII	Well developed	XVII–XX, rudimentary or absent	XVI–XXI	XVI–XX, wrinkled	XVI–XX, coarsely lobate
Spermathecae	About 2.4mm long, ampulla slender heart-shaped; duct short	Ampulla oval or pear-shaped, duct equal to or slightly shorter than ampulla	Ampulla ovoid	–	Ampulla peach-shaped, stalk straight, much shorter than ampulla	About 1.6mm long; ampulla heart-shaped, gradually slender duct as long as ampulla
Diverticulum	About 2.0mm long, terminal 4/5, swollen, S-shaped twisted seminal chamber	One-third to half of ampulla, slender stalk and a wider seminal chamber	Blunt ovioid, straight stalk	Shorter, seminal chamber ovoid and whitish	Shorter, seminal chamber rudimentary or absent, straight or slightly bent	As long as main spermathecal axis, slender, terminal 0.4 dilated into a band shaped chamber
Accessory glands	1 stalked accessory gland observed near the ental part of each spermatheca	–	Stalked, coelomic, bound down to parietes or retained within body wall	Invisible	round, stalked observed in spermathecal pores and male pores region	Invisible

#### Diagnosis.

Dimensions 55–86 mm by 2.5–2.8 mm at clitellum, 55–83 segments. First dorsal pore in 13/14. Setae numbering 24–26 at III, 34–36 at V, 34–36 at VIII, 32–36 at XX, 36–40 at XXV; 8–9 between male pores; setae between spermathecal pores numbering 9–12 at VI, 10–12 at VII, 12–13 at VIII. Four pairs of spermathecal pores in 5/6–8/9, eye-like. Four pairs of postsetal genital papillae in VI–IX, 0.20 circumference ventrally apart from each other. One pair of male pores in XVIII, each on the top of a central, round porophore surrounded by three or four rhombic ridges, with one presetal crescent indented-topped genital papilla medial of each male pore. Ampulla slender, heart-shaped; duct short. Diverticulum shorter than main pouch, terminal 4/5 slightly dilated into a swollen, S-shaped twisted seminal chamber. Prostate glands well developed.

#### Description.

**External characters**: Pre-clitellum, purple-brown pigment on dorsum, light purple-brown on ventrum. Post-clitellum, light purple-brown on dorsum, no pigment on ventrum. Clitellum taupe. Dimensions 76 mm by 27 mm at clitellum. 75 segments. Prostomium ½ epilobous. First dorsal pore in 13/14. Setae numbering 24 at III, 34 at V, 36 at VIII, 36 at XX, 40 at XXV; 8 between male pores; Setae between spermathecal pores numbering 11 at VI, 10 at VII, 13 at VIII. Setae formula AA = 1.2-2.0AB, ZZ = 1.4-2.0ZY. Clitellum annular, pale taupe, in XIV–XVI, setae not visible externally. Four pairs of spermathecal pores in 5/6–8/9, eye-like, 0.25 circumference ventrally apart from each other. Four pairs of postsetal genital papillae in VI–IX, 0.20 circumference ventrally apart from each other. One pair of male pores in XVIII, 0.25 circumference apart ventrally, each on the top of a central, round porophore surrounded by three or four rhombic ridges, with one presetal crescent indented-topped genital papilla in the center of each male pore region (Figure 2A). Single female pore in XIV.

**Internal characters.** Septa 5/6–7/8 thick and muscular, 10/11–12/13 slightly thickened, 8/9–9/10 absent. Gizzard ball-shaped, in VIII–X. Intestine enlarged distinctly from XVI onwards. Intestinal caeca paired in XXVII, simple, smooth, extending anteriorly to XXIV. Four pairs of esophageal hearts in X–XIII, the first pair very thin, the last three pairs developed. Ovaries in XIII. Four pairs of spermathecae in VI–IX, small, 2.4 mm long. Ampulla slender, heart-shaped; duct short. Diverticulum 2.0 mm long, slightly shorter than main pouch, terminal 4/5 slightly dilated into a swollen, S-shaped twisted seminal chamber (Figure 2B). One stalked accessory gland observed medial to each spermathecal duct. Holandric: two pairs of testis sacs in X–XI, separated from each other, developed. Two pairs of seminal vesicles in XI–XII, well developed. Prostate glands well developed, inserting in XVIII and extending from XVII–XXII, coarsely lobate, prostatic duct C-shaped, uniform thickness. No accessory glands observed.

#### Etymology.

The species is named after the crooked shape of its diverticulum.

#### Remarks.

*Amynthas tortuosus* sp. nov. is a comparatively small earthworm and shares some similarities to *A. carnosus* and *A. corticis*. All of them have genital markings both on the spermathecal and the male pore regions and share similar setal numbers. However, the body size of *A. tortuosus* sp. nov. is very distinct from others. The new species has roughly half the body size of *A. carnosus* and *A. corticis*, both in length and width. The first dorsal pore is in 13/14 and the distance between the spermathecal pores and male pores is less than those of *A. carnosus* and *A. corticis*. Moreover, *A. tortuosus* sp. nov. has long diverticulum with an S-shaped twisted seminal chamber and eight stalked accessory glands observed near the spermathecal duct, rather than a straight or slender stalk and an absence of accessory glands in *A. carnosus*.

We also compare the new species with *Amynthas homosetus* (Chen 1938) described from Hainan Island, which has a very similar distance between the male pores and spermathecal pores, and similar setal numbers. The differences between the two species are as follows: the body size of *A. tortuosus* is much smaller than *A. homosetus*; the size of prostate glands is larger in *A. tortuosus*; the shape of the seminal chamber is S-shaped twisted in *A. tortuosus*, while it is ovoid in *A. homosetus*; and several genital markings are present in the spermathecal pore region and male pore region in *A. tortuosus* sp. nov., while these markings are absent in *A. homosetus*.

The body size of *A. tortuosus* sp. nov. is similar to *Amynthas exiguus aquilonius* Tsai et al., 2001 described from Taiwan Island and *Amynthas stricosus* Qiu & Sun, 2012 described from Hainan Island. The first dorsal pore of the new species is in 13/14, but in 6/7 in *A. exiguus aquilonius*, and in 11/12 or 12/13 in *A. stricosus*. The new species has more closely spaced spermathecal pores than *A. exiguus aquilonius* and *A. stricosus*. Additionally, *A. exiguus aquilonius* has more genital papillae observed in spermathecal pores and male pores region than the new species. Accessory glands are observed in spermathecal pores and male pores region in *A. exiguus aquilonius*, but in the new species, accessory glands are only observed in spermathecal pores region. Furthermore, *A. stricosus* has a band shaped chamber, no genital papillae near spermathecal pores region, no accessory glands, and papillae observed in XVII, XIX and XX, but the new species has a S-shaped twisted seminal chamber, four pairs of genital papillae in spermathecal pores region, accessory glands near spermathecal duct, and no genital papillae observed in XVII, XIX and XX. Details of the comparison are showed in table 3.

### Amynthas shengtangmontis

Taxon classificationAnimaliaOpisthoporaMegascolecidae

Dong & Jiang sp. nov.

B443140B-C8AF-5425-BA14-A72F0DF6F86E

http://zoobank.org/6831C1EE-6B6B-4B4C-8C9F-4A9B903EBCB6

Figure 4 , Table 4

#### Material.

**Holotype**: 1 clitellate (C-GX201312-03A): China, Guangxi Zhuang Autonomous Region, Dayao Mountain National Nature Reserve (23.97299°N, 110.11106°E), 1210 m asl, black sandy soil in bamboo forest, 15 May 2013, JP Qiu, Y Hong, JB Jiang, LL Zhang, Y Dong legit. **Paratypes**: 4 clitellates (C-GX201312-03A): same data as for holotype.

#### Diagnosis.

Dimension 100–134 mm by 4.2–5.1 mm at clitellum, 116–138 segments. First dorsal pore in 12/13. Setae numbering 26–28 at III, 24–32 at V, 26–32 at VIII, 33–40 at XX, 35–42 at XXV; 8–11 between male pores. Setae between spermathecal pores numbering 11–15 at VI, 10–13 at VII, 12–16 at VIII. Four pairs of spermathecal pores in 5/6–8/9. Four pairs of postsetal genital papillae arranged in VI–IX, 0.25 circumference ventrally apart from each other. One pair of male pores in XVIII, each on the top of a large raised, round porophore, surrounded by two circular ridges, with one presetal indented-topped genital papilla medial of each porophore. First ampulla of the three pairs is heart-shaped, duct stalked, diverticulum as long as main chain, U-shaped twist in the middle, terminal 4/5 dilated into a club-shaped seminal chamber. Ampulla of the fourth pair elongate-oval, duct as long as 1/6 ampulla, diverticulum as long as main chain, U-shaped twist in the middle, terminal 1/3 dilated into a chili-shaped seminal chamber. One round, semitransparent accessory gland presents near the medial area of each spermatheca. The prostate glands are developed.

**Figure 4.**
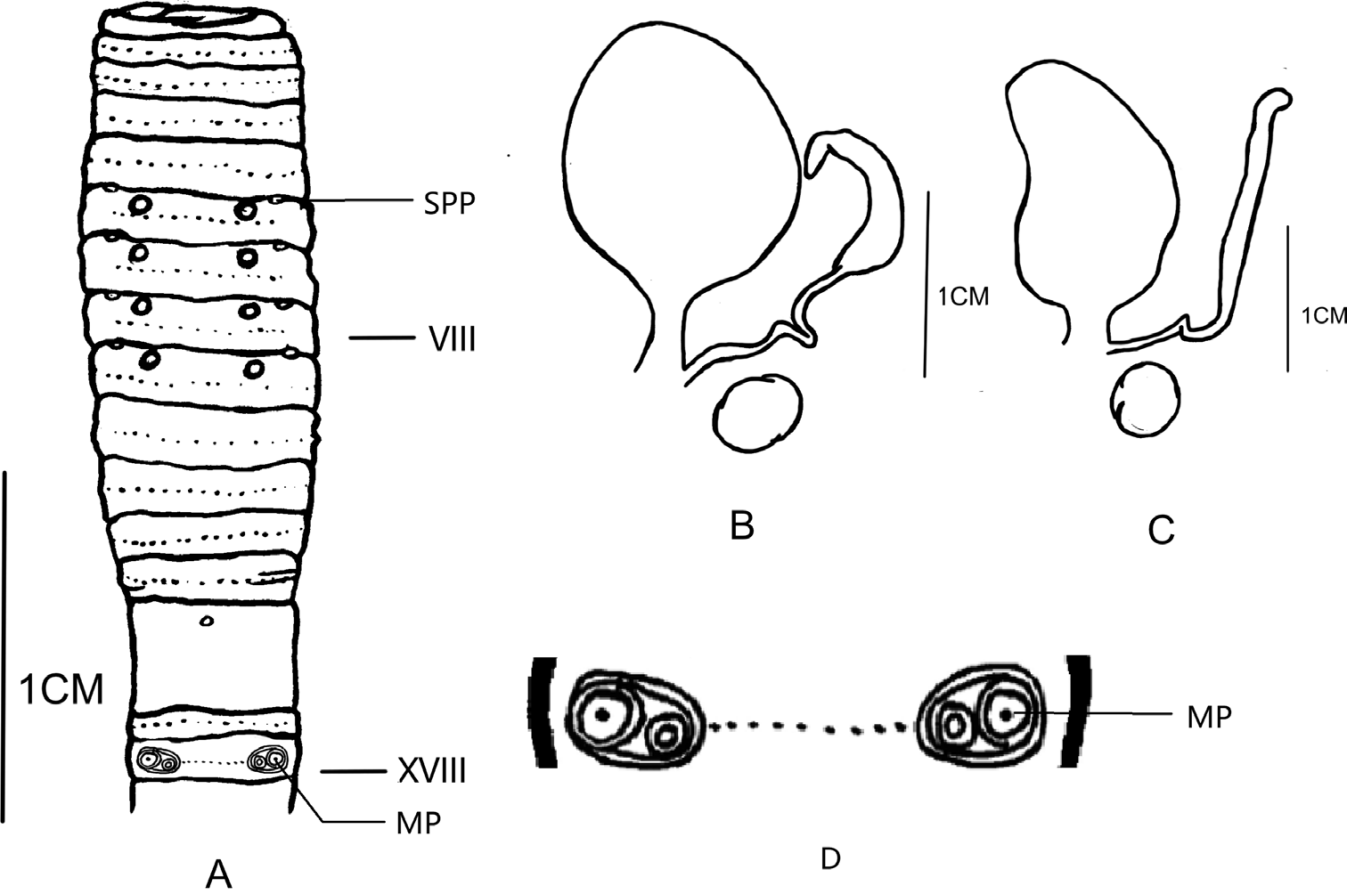
**A** Ventral view showing spermathecal pores, female pores and male pores of *Amynthas shengtangmontis* sp. nov. **B, C** spermathecae of *Amynthas shengtangmontis* sp. nov. **D** illustration of the details of the male pore region.

**Table 4.** A comparison of characters of *A. shengtangmontis shengtangmontis*, *A. shengtangmontis minusculus*, *A. carnosus*, *A. corticis*. and *A. pulvinus*.

**Characteristics**	***A. shengtangmontis shengtangmontis***	***A. shengtangmontis minusculus***	***A. carnosus* (Goto & Hatai, 1899)**	***A. corticis* (Kinberg, 1867)**	***A. pulvinus* Sun & Qiu, 2013**
Body length (mm)	100–134	75–83	110–340	45–170	93.5
Body width (mm)	4.2–5.1	3.0–3.2	4.0–9.0	3.0–6.0	3.4
Pigment					
dorsum	From brown to no	Purple brown	Dark brown or purple	Greenish brown	Buff
ventrum	From brown to no	No	Dark brown or purple	No	No
First dorsal pore	12/13	11/12	12/13	10/11 or 12/13, usually at 11/12	12/13
Clitellum locality	XIV–XVI	XIV–XVI	XIV–XVI	XIV–XVI, occasionally shorter	XIV–XVI
Spermathecal pores	4 pairs, in 5/6–8/9, 0.40C	4 pairs, in 5/6–8/9, 0.40C	4 pairs in 5/6–8/9 or 3 pairs in 6/7–8/9, 0.33C	4 pairs, 5/6–8/9, 0.33C	4 pairs, in 5/6–8/9, 0.33C
Male pores	Large raised, round, surrounded by 2 circular ridges, 0.44C	Raised, elliptic, surrounded by 6 circular ridges, 0.40C	Round or elliptic	Small, circular to transverse elliptical disc, 0.24–0.30C	Slightly elevated round, 0.33C
Papillae preclitellum	Four pairs of postsetal genital papillae arranged in VI–IX	Three pairs of postsetal genital papillae arranged in VI–VIII	8 papillae just overhead 8 spermathecal pores, 2 pairs preclitellar arranged on VIII and IX	Paired presetal and/or postsetal in some or all, near spermathecal pores	Invisible
Papillae postclitellum	2 presetal indented-topped genital medial of porophore	2 small indented-topped genital papillae medial of male pore	2 paired presetal genital on XVIII and IX, 1 pair postsetal genital on XVIII	Present or absent, occasionally one or more near male pore	Rectangle-shaped, on 17/18–18/19
Prostate glands	XV–XXII	XVI–XXI	Well developed	XVII–XX, rudimentary or absent	XVII–XX
Spermathecae	About 2.2–3.0mm long, ampulla of the first three pairs heart-shaped, duct stalk. Ampulla of the forth pair long-oval, duct as long as 1/6 ampulla	About 2.2–2.7mm long, duct as long as 1/2 ampulla	Ampulla oval or pear-shaped, duct equal to or slightly shorter than ampulla	Ampulla ovoid	About 2.4mm long, ampulla slender heart-shaped; duct short
Diverticulum	As long as main chain, U-shaped twisted in middle, terminal 4/5, club-shaped seminal chamber of the first three pairs and terminal 1/3 dilated into a chilli-shaped seminal chamber of the forth pair	As long as main chain, terminal 1/2, long club-shaped seminal chamber	One-third to half of ampulla, slender stalk and a wider seminal chamber	Blunt ovioid, straight stalk	Shorter, terminal 1/5, small ovoid plump seminal chamber
Accessory glands	1 round semitransparent accessory gland present near the medial area of each spermatheca, 2 stalk accessory glands observed near the medial of the distal part of the prostatic duct	6 semitransparent elliptic accessory glands observed near the distal part of the last three pairs spermathecae	–	Stalked, coelomic, bound down to parietes or retained within body wall	A pair, cling to body wall, irregular in shape, and extended from XVII–XIX

#### Description.

**External characters**: Pigment from brown to no pigment on dorsum, from light brown to no pigment on ventrum. Dimensions 102 mm by 4.5 mm at clitellum, 117 segments. Prostomium ½ epilobous. First dorsal pore in segments 12/13. Setae numbering 26 at III, 26 at V, 29 at VIII, 36 at XX, 37 at XXV; 9 between male pores. Setae between spermathecal pores numbering 13 at VI, 12 at VII, 14 at VIII. Setal formula: AA = 1.0-1.4AB, ZZ = 2.0-2.2ZY. Clitellum annular, in XIV–XVI, setae not visible externally. Four pairs of spermathecal pores in 5/6–8/9, 0.40 circumference apart ventrally. Four pairs of genital papillae on VI–IX, 0.25 circumference ventrally apart from each other. One pair of male pores in XVIII, 0.40 circumference ventrally apart from each other, each on the top of a larger raised, round porophore, surrounded by two circular ridges, with one presetal indented-topped genital papilla medial of each porophore (Figure 3A). Singled female pore in XIV, pale grey.

**Internal characters.** Septa 5/6–7/8 thick and muscular, 10/11–11/12 slightly thickened, 8/9–9/10 absent. Gizzard bucket-shaped, wider below than above, in VIII–X. Intestine enlarged distinctly from XV. Intestinal caeca paired in XXVII, simple, smooth, extending anteriorly to XXI. Esophageal hearts in X–XIII. Ovaries in XIII, four pairs of spermathecae in VI–IX, 2.2–3.0 mm long. Spermathecae of two shapes: ampulla of the first three pairs heart-shaped, duct stalked, diverticulum as long as main chain, U-shaped twist in the middle, terminal 4/5 dilated into a club-shaped seminal chamber. The ampulla of the fourth pair elongate-oval, duct as long as 1/6 ampulla (Figure 3C), and diverticulum as long as main chain, U-shaped twisted in the middle, terminal 1/3 dilated into a chili-shaped seminal chamber (Figure 3B). One round semitransparent accessory gland presents near the medial area of each spermatheca. Holandric: two pairs of testis sacs in X–XI, separated from each other, well developed. Two pairs of seminal vesicles, in XI–XII, well developed. Prostate glands developed, thick, inserting in XVIII and extending from XV to XXII, coarsely lobate, prostatic duct U-curved, slightly thicker at the ental part. Two stalked accessory glands near the medial area of the distal part of the prostatic duct.

#### Etymology.

The species is named after the name of the collection site Shengtang Mountain, a famous peak of the Dayao Mountain National Nature Reserve.

#### Remarks.

*Amynthas shengtangmontis* sp. nov. keys to the *corticis*-group in Sims and Easton (1972). In terms of morphology, it is closely related to *A. carnosus*, *A. corticis*, and *Amynthas pulvinus* Sun & Jiang, 2013 (described from Hainan Island). Body size, body pigment, setal numbers, the first dorsal pore location, and simple intestinal caeca are similar among the four species.

In contrast, distance between the spermathecal pores and the male pores of the new species is 0.40C body circumference compared with 0.33C in *A. carnosus*, *A. corticis*, and *A. pulvinus*. In addition, there are eight postsetal genital markings on VI–IX in the new species, but the markings are present on V–VIII in *A. carnosus*, and there are more than two pairs of markings on VIII and IX in *A. carnosus*. The porophore of the new species is large, raised, round, and surrounded by two circular ridges, whereas the porophore is small in *A. corticis* and no genital markings apparent on the spermathecal pore region in *A. pulvinus*. Moreover, the new species has two different shapes of spermathecae, heart-shaped ampulla and diverticulum with club-shaped seminal chamber; and long-oval ampulla and diverticulum with chili-shaped seminal chamber, which are very different from those in the other species (Table 4).

### Amynthas shengtangmontis minusculus

Taxon classificationAnimaliaOpisthoporaMegascolecidae

Dong & Law subsp. nov.

CA5B4CF4-1874-597C-A31D-0639C517E89E

http://zoobank.org/AE048B96-5A37-4C48-A734-22EA11119010

Figure 5 , Table 4

#### Material.

**Holotype**: 1 clitellate (C-GX201316-02A): China, Guangxi Zhuang Autonomous Region, Dayao Mountain Nature Reserve (24.16658°N, 110.24313°E), 1285 m asl, black sandy soil under bryophytes beside road, 16 May 2013, JP Qiu, Y Hong, JB Jiang, LL Zhang, and Y Dong legit. **Paratypes**: 6 clitellate (C-GX201316-02B): same date as for holotype.

#### Diagnosis.

Dimensions 75–83 mm by 3.0–3.2 mm at clitellum, 75–87 segments. First dorsal pore in 11/12. Setae numbering 21–26 at III, 18–21 at V, 27–32 at VIII, 29–34 at XX, 36–40 at XXV; 5–7 between male pores; Setae between spermathecal pores numbering 9–11 at VI, 10–12 at VII, 10–12 at VIII. Four pairs of spermathecal pores in 5/6–8/9. Three pairs of postsetal genital papillae arranged in VI–VIII, 0.13 circumference apart ventrally. One pair of male pores in XVIII, each on the top of a raised, elliptic porophore surrounded by six circular ridges, with one small indented-topped genital papilla medial of each male pore. Ampulla heart-shaped; stout duct as long as 1/2 ampulla. Diverticulum as long as main pouch, terminal 1/2 dilated into a long club-shaped seminal chamber. Prostate glands developed.

**Figure 5.**
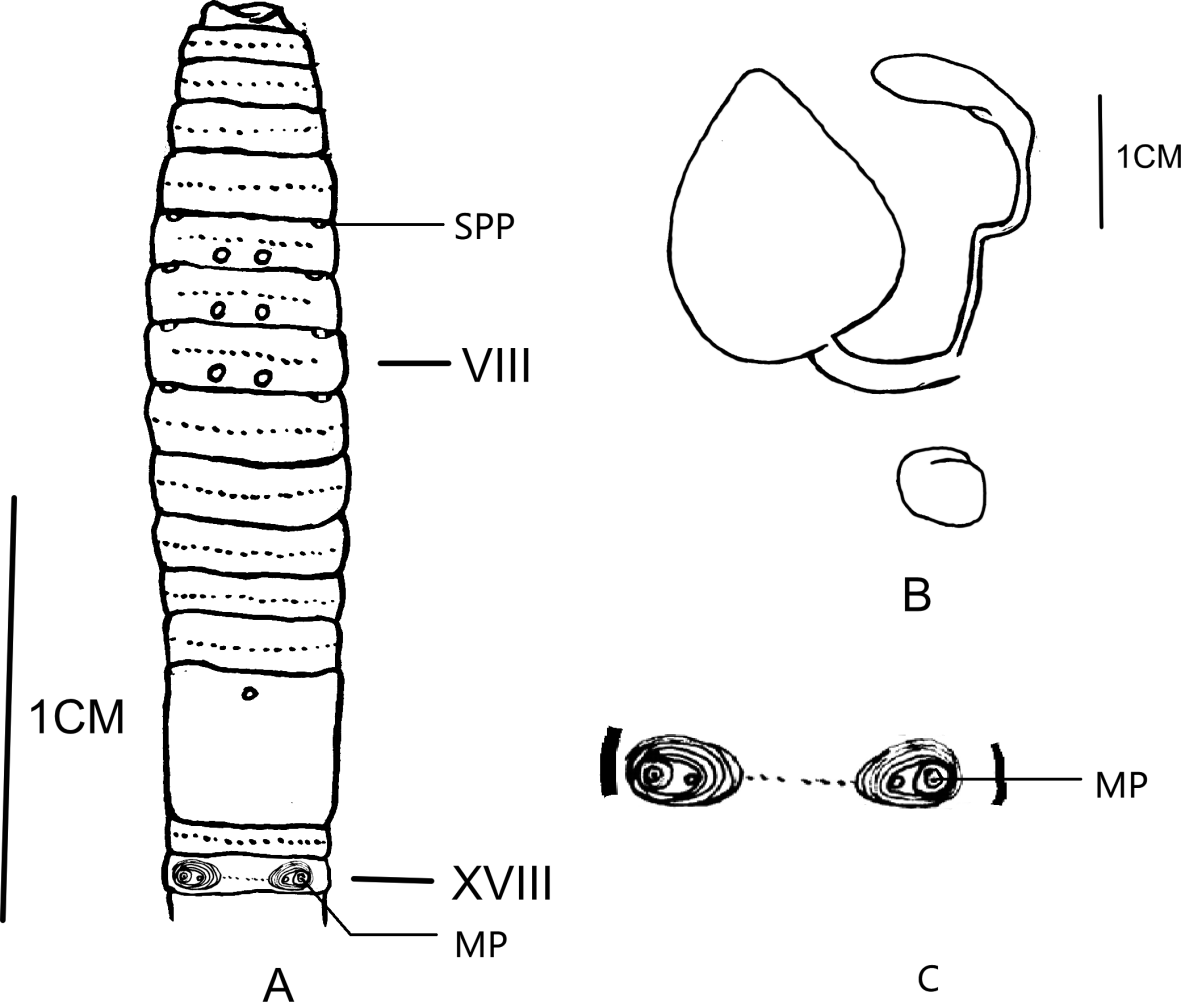
**A** Ventral view showing spermathecal pores, female pores and male pores of *Amynthas shengtangmontis minusculus* subsp. nov. **B** spermathecae of *Amynthas shengtangmontis minusculus* subsp. nov. **C** illustration of the details of the male pore region.

#### Description.

**External characters**: Purple brown pigment on dorsum, no pigment on ventrum. Dimensions 83 mm by 3.2 mm at clitellum, 87 segments. Prostomium ½ epilobous. First dorsal pore in 11/12. Setae numbering 26 at III, 21 at V, 32 at VIII, 34 at XX, 38 at XXV; 6 between male pores; setae between spermathecal pores numbering 10 at VI, 11 at VII, 12 at VIII. Setal formula AA = 1.2-1.4AB, ZZ = 1.4-1.8ZY. Clitellum annular in XIV–XVI, setae not visible externally. Four pairs of spermathecal pores in 5/6–8/9, 0.40 circumference ventrally apart from each other. Three pairs of postsetal genital papillae arranged in VI–VIII. One pair of male pores in XVIII, 0.40 circumference apart ventrally, each on the top of a raised, elliptic porophore surrounded by six circular ridges, with one small indented-topped genital papilla in the center of each male pore (Figure 4A). Single female pore in XIIV.

**Internal characters.** Septa 5/6–7/8, thick and muscular, 10/11–11/12 slightly thickened, 8/9–9/10 absent. Gizzard bucket-shaped, wider below than above, in IX–X. Intestine enlarged distinctly from XV. Intestinal caeca paired in XXVII, simple, smooth, extending anteriorly to 1/2 XXIV. Esophageal hearts in X–XIII. Ovaries in XIII, four pairs of spermathecae in VI–IX, heart-shaped, 2.2–2.7mm long, duct as long as 1/2 ampulla. Diverticulum as long as main pouch, terminal 1/2 dilated into a long club-shaped seminal chamber. Six semitransparent elliptical accessory glands observed near the ental part of the last three pairs spermathecae (Figure 4B). Holandric: two pairs of testis sacs, separated from each other, in X–XI, oval, the first pair extremely developed. Two pairs of seminal vesicles, in XI–XII, developed. Prostate glands, thick, inserting in XVIII and extending from XVI to XXI, developed, coarsely lobate; prostatic duct U-curved, long, slightly thicker at the ental part.

#### Remarks.

We compare *A. shengtangmontis minusculus* subsp. nov. with *A. shengtangmontis shengtangmontis* and find that they share similar characters of spermathecal pores, male pores, intestinal caeca, and prostate glands. However, there is a level of difference between them. For instance, *A. shengtangmontis minusculus* subsp. nov. has a smaller body size, fewer and more closely spaced genital papillae, longer spermathecal duct, accessory glands only observed in the spermathecal area. On the other hand, the first dorsal pore of *A. shengtangmontis minusculus* subsp. nov. is located in 11/12 compared to in 12/13 in *A. shengtangmontis shengtangmontis*. The pairwise distance of COI between *A. shengtangmontis shengtangmontis* and *A. shengtangmontis minusculus* subsp. nov. is 10.7%-11.4%, which is acceptable to differentiate subspecies.

#### Etymology.

The subspecies is named after its small body size, compared to the nominate species.

## Molecular results

In addition to the morphological comparison between the species, we also compared the COI gene sequences of the three proposed new species and one subspecies and the results of the pairwise distances of COI, ranging from 10.7%-25.2%% are shown in Table 5. Studies show that pairwise distances of COI of interspecies in the same genus are 17–23% (Sun 2013), 16–23% (Huang et al. 2007), 15–16% (Admassu et al. 2006), 16–22% (Novo et al. 2009), and 15–28% (Chang et al. 2008). In general, pairwise distances between three new species and the other eighteen *corticis*-group species are greater than 14.7%. Together with the different morphological characters of each, we could conclude that *A. maximus*, *A. tortuosus*, *A. shengtangmontis shengtangmontis*, and *A. shengtangmontis minusculus* are different from the previously described species and each other. Since the pairwise distance between *A. shengtangmontis shengtangmontis* and *A. shengtangmontis minusculus* is 10.7%-11.4%, which is more than 1% and less than 15%, by definition we conclude that both subspecies are valid.

**Table 5.** Percentage of pairwise distances obtained for the sequences of COI genes in *Amynthas* species.

	1	2	3	4	5	6	7	8	9	10	11	12	13	14	15	16	17	18	19	20	21	22	23	24
S1 HT																								
S2 HT	19.2%																							
S2 PT	19.2%	0.0%																						
S2 PT	19.2%	0.0%	0.0%																					
S3 HT	16.4%	15.7%	15.7%	15.7%																				
S4 HT	16.7%	14.7%	14.7%	14.7%	10.7%																			
S4 PT	16.9%	15.4%	15.4%	15.4%	11.4%	0.2%																		
*A. fuscatus*	15.9%	17.4%	17.4%	17.4%	20.4%	17.4%	17.0%																	
*A. pulvinus*	20.0%	18.2%	18.2%	18.2%	19.7%	18.4%	18.8%	18.6%																
*A. robustus*	18.9%	16.9%	16.9%	16.9%	17.8%	18.0%	18.0%	17.1%	22.7%															
*A. corticis*	15.6%	18.0%	18.0%	18.0%	18.2%	18.7%	18.8%	18.0%	16.3%	18.0%														
*A. carnosus*	19.8%	17.4%	17.4%	17.4%	20.2%	20.0%	19.9%	18.3%	18.7%	16.7%	16.7%													
*A. micronarius*	20.7%	19.8%	19.8%	19.8%	19.9%	19.5%	20.1%	21.0%	19.1%	20.9%	17.5%	21.2%												
*A. alexandri*	24.3%	21.3%	21.3%	21.3%	23.7%	22.4%	22.0%	21.3%	21.5%	20.7%	21.5%	20.8%	22.6%											
*A. andersoni*	23.2%	20.1%	20.1%	20.1%	23.0%	21.0%	20.9%	18.9%	21.2%	19.6%	17.3%	18.4%	20.9%	23.6%										
*A. comptus*	21.9%	19.2%	19.2%	19.2%	20.7%	19.4%	20.2%	19.1%	21.2%	19.6%	18.5%	20.0%	20.5%	23.5%	19.2%									
*A. exiguus*	18.2%	18.0%	18.0%	18.0%	18.1%	18.5%	18.7%	18.2%	18.6%	18.7%	18.5%	19.1%	18.3%	24.2%	21.3%	22.1%								
*A. formosae*	21.4%	21.0%	21.0%	21.0%	19.3%	21.9%	22.0%	23.2%	19.8%	22.7%	20.3%	22.7%	20.8%	20.5%	24.4%	24.1%	23.5%							
*A. longicauliculatus*	20.2%	19.5%	19.5%	19.5%	21.6%	21.8%	22.1%	19.6%	21.5%	21.3%	19.8%	22.6%	21.7%	22.9%	18.3%	18.1%	20.2%	23.9%						
*A. mirifius*	17.6%	16.6%	16.6%	16.6%	18.7%	17.1%	17.1%	16.1%	16.7%	17.9%	15.3%	17.4%	20.5%	19.2%	20.7%	19.8%	18.9%	21.3%	19.7%					
*A. szechuanensis*	22.5%	20.4%	20.4%	20.4%	22.6%	20.4%	20.5%	19.3%	22.4%	21.8%	18.2%	19.8%	21.5%	24.7%	19.3%	18.9%	20.0%	24.0%	18.3%	21.4%				
*A. mediocus*	19.4%	19.4%	19.4%	19.4%	19.4%	23.8%	23.6%	18.0%	19.8%	19.1%	18.5%	20.5%	21.4%	25.2%	22.5%	18.7%	18.2%	24.2%	20.9%	18.8%	22.5%			
*A. wulinensis*	20.2%	18.3%	18.3%	18.3%	21.8%	18.4%	18.4%	19.4%	20.9%	20.2%	17.8%	19.7%	18.2%	25.1%	20.7%	21.5%	20.4%	23.0%	23.2%	19.9%	19.2%	19.7%		
*A. yunlongensis*	19.7%	18.6%	18.6%	18.6%	18.4%	16.8%	17.3%	19.2%	18.0%	17.4%	17.8%	17.9%	20.3%	21.3%	22.2%	19.8%	17.1%	21.0%	20.0%	17.0%	16.2%	20.3%	20.5%	
*A. stricosus*	16.3%	15.0%	15.0%	15.0%	18.2%	16.6%	16.4%	13.1%	18.4%	17.1%	16.1%	17.2%	20.8%	18.4%	17.3%	17.6%	21.7%	18.8%	18.4%	19.5%	20.8%	18.1%	16.5%	21.1%

Notes: S1 represent *A. maximus*, S2 represent *A. tortuosus*, S3 represent *A. shengtangmontis shengtangmontis*, S4 represent *A. shengtangmontis minusculus*

## Supplementary Material

XML Treatment for Amynthas maximus

XML Treatment for Amynthas tortuosus

XML Treatment for Amynthas shengtangmontis

XML Treatment for Amynthas shengtangmontis minusculus
